# Look and ye shall hear: Selective auditory attention modulates the audiovisual correspondence effect

**DOI:** 10.1177/20416695221095884

**Published:** 2022-05-23

**Authors:** Armina Janyan, Yury Shtyrov, Ekaterina Andriushchenko, Ekaterina Blinova, Olga Shcherbakova

**Affiliations:** Research Center for Cognitive Science, 54526 New Bulgarian University, Sofia, Bulgaria; Center of Functionally Integrative Neuroscience, Institute for Clinical Medicine, 1006 Aarhus University, Aarhus, Denmark; Laboratory of Behavioural Neurodynamics, 408258 Saint Petersburg State University, Saint Petersburg, Russia; Laboratory of Behavioural Neurodynamics, 408258 Saint Petersburg State University, Saint Petersburg, Russia; Department of General Psychology, Faculty of Psychology, Saint Petersburg State University, Saint Petersburg, Russia

**Keywords:** audiovisual correspondence, selective attention, RT

## Abstract

One of the unresolved questions in multisensory research is that of automaticity of consistent associations between sensory features from different modalities (e.g. high visual locations associated with high sound pitch). We addressed this issue by examining a possible role of selective attention in the audiovisual correspondence effect. We orthogonally manipulated loudness and pitch, directing participants’ attention to the auditory modality only and using pitch and loudness identification tasks. Visual stimuli in high, low or central spatial locations appeared simultaneously with the sounds. If the correspondence effect is automatic, it should not be affected by task changes. The results, however, demonstrated a cross-modal pitch-verticality correspondence effect only when participants’ attention was directed to pitch, but not to loudness identification task; moreover, the effect was present only in the upper location. The findings underscore the involvement of selective attention in cross-modal associations and support a top-down account of audiovisual correspondence effects.

We live in a rich multimodal environment, constantly processing information coming from different sources and modalities, integrating it and making sense of the entire complexity of diverse inputs. Ample research evidence suggests that multimodal sensory processing promotes interactions between the sensory systems (e.g., Spence, 2011; Störmer, 2019), and the diverse information that bombards us may, in fact, help us to perceive and understand. Whereas there are many different cross-modal combinations of features that interact (Spence, 2011; Parise, 2016), we focus here on two particularly important modalities—vision and audition—and investigate audiovisual interactions between specific stimulus features: spatial position, pitch, and loudness.

A body of behavioural research has successfully demonstrated that visuo-spatial and auditory (pitch) information tend to interact. Congruent trials (high pitch coupled with higher position in space) seem to speed up processing (e.g., Evans & Treisman, 2010; Jonas, Spiller, & Hibbard, 2017; Evans, 2020; see also reviews by Spence, 2011; and Parise, 2016), compared to incongruent trials (high pitch coupled with lower position in space). Parise, Knorre, & Ernst (2014) demonstrated that this cross-modal correspondence comes from the statistics of natural auditory scenes (sounds that come from above—e.g., bird cries—often have more energy at the higher end of acoustic spectrum). Thus, sound localisation and sound frequency are linked via natural statistical mapping; this widely studied cross-modal correspondence between *pitch and vertical space* appears to be a rather stable effect.

The situation is different with *loudness*. Mostly, the coupling between loudness and visual stimulus properties have been addressed in association with brightness (loud-bright, soft-dark; cf. Spence, 2011). Very few studies have directly examined the relationship between loudness and visual position, one of the most obvious combinations being loud-up/soft-down. This type of interaction effect does not seem stable and is probably semantically mediated (Puigcerver, Rodríguez-Cuadrado, Gómez-Tapia, & Navarra, 2019). Moreover, there may be no explicit experiential or sensory/acoustic basis for such a relationship. Nevertheless, Eitan, Schupak, & Marks (2008) have found a cross-modal correspondence effect by manipulating loudness and vertical motion. Given the scarcity of loudness-verticality data and different experimental paradigms applied (e.g., moving dots presented for 1 s in the above Eitan et al., 2008 study, or learned colour-sound associations coupled with mouse tracking in Puigcerver et al., 2019) as well as the lack of stable results, it seems theoretically important to test the relationship in a paradigm that is more relevant to the conditions in which multisensory integration occurs. Attesting loudness-vertical space correspondence in a balanced fashion was therefore one of the goals of the present study.

Even though cross-modal correspondence effect is widely researched, there seem to be no consensus concerning its underlying mechanisms. One of the unresolved theoretical questions is the question of the role of attention in the effect. A number of studies argue that correspondence takes place in a parallel and automatic fashion (Evans & Treisman, 2010) and is independent of selective attention (Evans, 2020), while other studies suggest that the origin of the correspondence effect could be top-down and attentional in nature (Koelewijn et al., 2010; Talsma, 2015). One important distinction between studies that support an attentional account and studies that support an automatic mechanism of cross-modal correspondence effect pertains to the methodological approach. Most behavioural studies that support an attentional account are based on a cueing paradigm which allows enough time between a cue and a target for endogenous process to occur as well (Getz, & Kubovy, 2018; Chiou, & Rich, 2012), whereas most studies that support automaticity use simultaneous brief presentations of visual and auditory stimuli (Evans, & Treisman, 2010; Evans, 2020, Experiments 1 and 3).

Evans (2020) argues that although Evans and Treisman (2010) suggested that the cross-modal effect is automatic, there is a possibility that the Evans and Treisman's (2010) study did not use tasks that would fully engage attentional resources so that there were enough resources to process the distractors (task-irrelevant dimensions) as well. Evans (2020) based her argument on Load Theory of Attention and Cognitive Control (Lavie, & Dalton, 2014; see also Lavie, 2005). The authors of the theory state that low perceptual demands would allow allocation of available resources towards task-irrelevant stimuli while high perceptual load that fully engages attention would decrease distractor interference. On the other hand, according to theories of automaticity, a fully automatic process should be insensitive to resource allocation, task demands, or goals (Moors, & De Houwer, 2006). To examine whether the cross-modal effect depends on selective attention, Evans (2020) conducted experiments on audiovisual correspondence presenting the audio (pitch) and visual (spatial location) stimuli simultaneously (In Experiments 1 and 3), asking participants to discriminate between sounds of musical instruments or orientation of visual grating. The aim was to test if the cross-modal correspondence effect would be influenced by increased attentional demands. The experiments examined perceptual load (low vs. high; Experiment 1), visual search (easy vs. difficult letter discrimination; Experiment 2), and divided attention (dual vs. single task; Experiment 3). Evans (2020) argued that if the cross-modal correspondence effect would not be affected by the different task demands it would mean that the effect is automatic. The results showed no task demand influence on the cross-modal correspondence effect. To argue for the automaticity of the effect, the study applied the four criteria of automaticity defined by Moors and De Houwer (2006) which are applicable to our experimental goals as well. They defined automaticity as an “umbrella” term under which there are four critical features: goal-independence, non-consciousness, load-insensitivity, and speed. We adopted the same approach and developed it further by overtly directing participants’ attention to one of the key auditory properties: pitch or loudness. In Evans (2020) only one of the visual or auditory features was relevant to the cross-modal correspondence (visual position and pitch), while task-related features (grating and musical instrument) were irrelevant to the expected interaction. On one hand, this approach strengthens the results, showing task and load insensitivity of the effect with an indirect task, on the other hand, however, it is possible that directing attention towards effect-irrelevant features did not engage full attention to all perceptual features (Lavie, 2005) thus allowing the ‘attention-free’ features to ‘escape’ and interact. Therefore, we slightly modified the Evans (2020) approach, presenting a task that directed attention to an effect-related feature and increasing perceptual load by introducing two potentially effect-relevant auditory features (pitch and loudness), leaving visual modality task-irrelevant. Note that the pitch-spatial location effect is widely researched, is stable and reliable (Spence, 2011) but the putative loudness-visual location effect is not, as mentioned above.

The main aim of the experiment was to test the attentional account of the cross-modal correspondence effect. We examined whether the attentional shift to different sound attributes would lead to a change in the cross-modal correspondence effect. If the process is totally automatic, we should observe no task effect, whereas involvement of top-down attention would modulate the correspondence effect depending on the attended feature. In this case the results would support a novel attentional framework that suggests that multisensory processing is modulated by both, top-down and bottom-up processes (Tang, Wu, & Shen, 2016). We focused on the pitch-location correspondence effect since it is a well-researched and reliable effect (Spence, 2011) whereas the loudness-location is not, as discussed above. Nevertheless, in the case of a stable loudness-location correspondence effect we would expect that convergent auditory features (e.g., high pitch and loud sound) would contribute to a stronger cross-modal correspondent effect compared to divergent features (e.g., high pitch and soft sound). Lastly, we aimed to examine the cross-modal effect separately for each spatial position. Most research (Evans, 2020; Getz, & Kubovy, 2018; Chiou, & Rich, 2012) on cross-modal correspondence uses the so-called ‘congruency effect’ dividing trials into ‘congruent’ (e.g., high pitch-upper position and low pitch-bottom position) and ‘incongruent’ conditions, masking the information about possible asymmetries across conditions. We consider it important to test whether the effect is symmetrical or whether different spatial/sound conditions differentially contribute to it due to perceptual and attentional asymmetries in visual vertical dimension (Jóhannesson, Tagu, & Kristjánsson, 2018). For instance, upper visual field has advantage in object detection and identification (Jóhannesson et al., 2018; Thomas, & Elias, 2011), and saccades are made faster to upper visual field (Greene, Brown, & Strauss, 2019) in comparison to lower visual field. In addition, a proper control/neutral condition would be needed to clarify if the effect is inhibitory or facilitatory. We therefore included such a condition here in visual, task-unrelated modality, where a screen-centre position could be used as a neutral stimulus.

## Method

### Participants

Fifty volunteers (38 females; M_age_ = 22.7 years, SD_age_ = 4.1 years) were recruited and participated in the experiment. All of them were right-handed, had normal or corrected-to-normal vision and normal hearing abilities. The research protocol was approved by the Research Ethics Committee of Saint Petersburg State University. All participants provided written informed consent prior to the participation and were informed about the general purpose of the study and the experimental procedure.

### Stimuli, Design, and Procedure

The study applied a 2 (Pitch: High vs. Low) × 2 (Loudness: Loud vs. Soft) × 3 (Visual stimulus position: Up vs. Centre vs. Down) × 2 (Task: Pitch identification vs. Loudness identification) within-subject design.

Sinusoidal tones varying in loudness (75 or 85 dB) and pitch (1000 or 2000 Hz) were composed using Audacity v.2.3 software (Audacity Team). To make sure that subjective loudness level per pitch height was controlled, the stimuli were checked against recent standards of loudness contour (Suzuki, & Takeshima, 2004), confirming that the pitch sounds were practically of the same subjective loudness (i.e., 1000 and 2000 Hz at 85 dB were perceived as equally loud). The sounds were presented binaurally through CX 300-II Precision headphones (Sennheiser).

Visual stimulus was a black circle with a diameter of 1.5 сm which corresponded to a visual angle of about 1.5 degrees. The circle was presented against a light-grey background (180, 180, 180 in RGB colour space) on a computer screen (ASUS ROG PG278Q, 144Hz refresh rate, diagonal: 27’’; screen resolution: 2560 × 1440 pixels). Both ‘down’ and ‘up’ positions deviated from the centre of the screen by 600 pixels corresponding to approximately 13 degrees from the screen centre to the circle centre. Participants were positioned at approximately 60–65 cm distance from the computer screen.

Tasks were blocked and the task sequence was counterbalanced across participants. Both tasks (see task details below) included a familiarisation part, practice part, and the main experimental part. Each part was preceded by an instruction presented on the computer screen. Familiarisation part included 12 trials that consisted of sounds (with 100 ms duration) presented together with their printed labels in the centre of the screen (“loud” or “soft”; “high” or “low”) for 2000 ms. Practice included 2 trials per condition (24 trials per task) followed by experimental part with 30 trials per condition (360 trials per task).

Participants were tested individually, in a sound-proof room. Each trial in the task was structured as follows. After fixation cross, presented on the screen for 500 ms, a sound was presented simultaneously with the visual stimulus for 100 ms. Participants were required to look at the screen and to use one finger of their left hand to identify, as fast and accurately as possible, either high/low pitch in the pitch block, or loud/soft sounds in the loudness block. They had to respond within 1000 ms, pressing a corresponding button on an RB-740 response pad (Cedrus; timing precision ± 5ms). The intertrial interval was 1000 ms. Two buttons of the response pad were used (the distance between buttons was approximately 30 mm).

**Figure 1.**
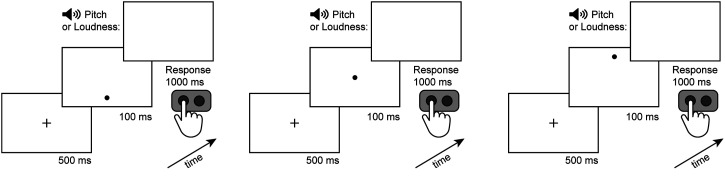
Examples of trials with different circle positions (down, centre, and up). Trial sequence: Fixation cross appeared for 500 ms, then auditory and visual stimuli were presented simultaneously for 100 ms, followed by a response window of 1 sec or until a response was given. Participants were required to look at the screen and to identify either the pitch (high/low) or loudness (loud/soft) of the presented sound, and press the corresponding button.

The sounds were the same in each block (randomised anew for every participant), only the instructions differed. The need to look at the screen was repeatedly stressed during the instruction, moreover, participants’ gaze position was monitored by a camera in the experimental room. The response mapping to specific button was counterbalanced across tasks and participants. The experiment took about one hour. Upon request, participants could have a short break (5–7 min) between the task blocks. NBS Presentation® software v.21.0 (Neurobehavioural Systems) was used to control stimulus presentation and response collection (Figure 1).

### Statistical Analysis

Data of nine participants were excluded from further analysis based on their low compliance (low accuracy together with no response (< 80% overall). A post-hoc power analysis indicated that with this sample size (41 participants) our experiment had sufficient power to detect even a small effect (power = 0.97 for an effect size f = 0.25). The post-hoc power analysis was conducted using G*Power 3.1.9.6 (Faul, Erdfelder, Lang, & Buchner, 2007).

**Figure 2.**
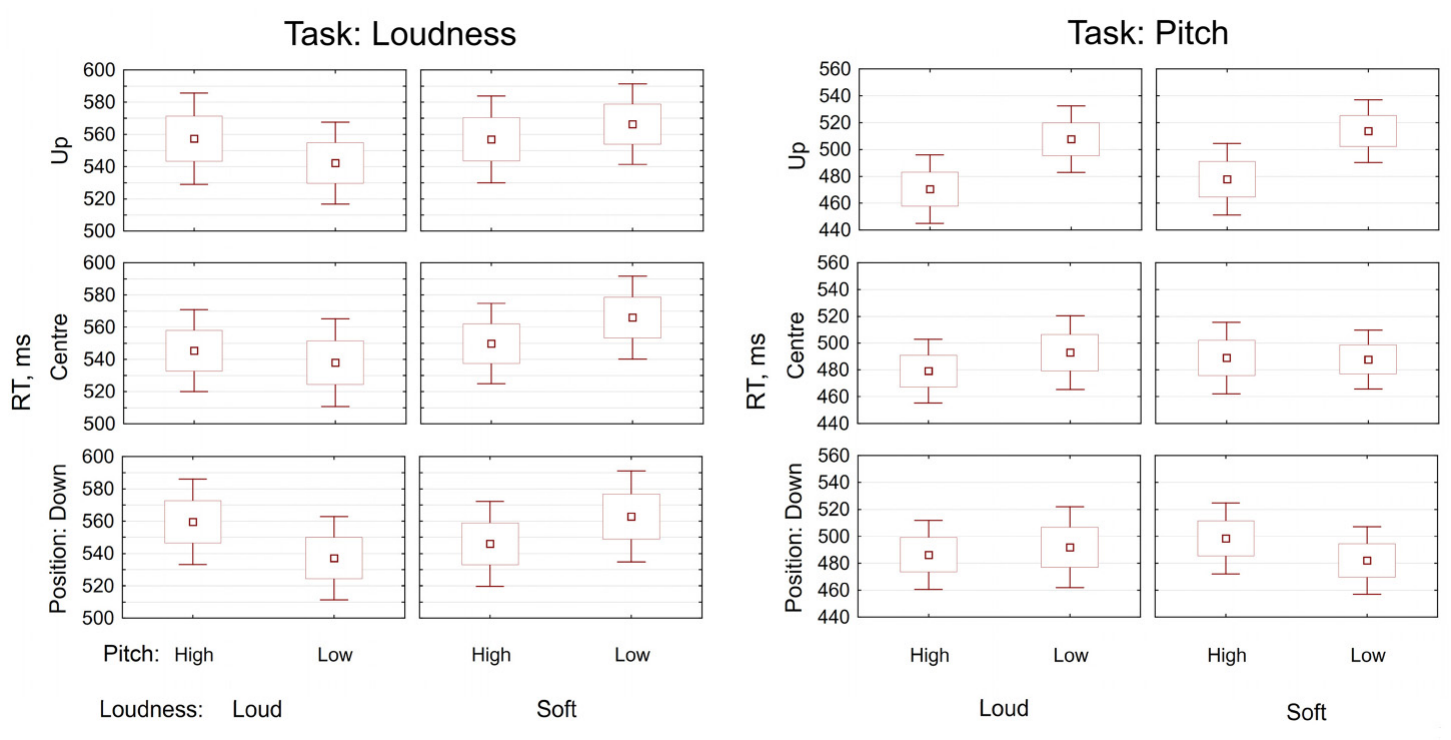
Data visualisation, descriptive. Reaction times per condition, separately for each task (Left panel: Loudness task, Right panel: Pitch Task). Upper part of the x-axis indicates Pitch levels, and lower part—Loudness levels. Dot position levels are indicated on the y-axis. *Note.* Dots denote Means, Boxes—Mean ± SE, and vertical bars—Mean ± 95% CI*.*

From the remaining data of 41 participants erroneous (2.3%) and missed responses (4.9%) were excluded. Reaction time (RT) was measured from the stimulus offset. Outliers with RT outside of ± 2SD were removed based on individual subject variance per condition (4.4%). The single-trial RT data were then averaged for each subject and condition and the resulting values were entered into a repeated-measures ANOVA (rmANOVA) with within-subject factors Pitch (High/Low), Loudness (Loud/Soft), Visual position (Up/Centre/Down) and Identification Task (Pitch/Loudness). Bonferroni-corrected post-hoc tests were applied where appropriate. Table 1 and Figure 2 present descriptive statistics per condition.

**Table 1.** Reaction times: means (M) and standard deviations (SD) per condition, ms. Within each cell, descriptive statistics of tasks is separated by a forward slash (Pitch/Loudness).

Task	Loudness/Position	High Pitch			Low Pitch		
		Up M(SD)	Centre M(SD)	Down M(SD)	Up M(SD)	Centre M(SD)	Down M(SD)
Pitch/ Loudness	Loud	471(81)/557(90)	479(75)/545(81)	486(81)/560(84)	508(78)/542(81)	493(87)/538(86)	492(95)/537(82)
	Soft	478(85)/557(85)	48­9(85)/550(79)	498(83)/546(83)	514(74)/566(79)	488(70)/566(81)	482(79)/563(89)

## Results

RmANOVA revealed three significant main effects. A main effect of task (F(1,40) = 44.31, p < 0.001, η_p_^2^ = 0.53) showed overall slower identification of loudness features (552 ms) than those of pitch (490 ms). A main effect of loudness (F(1,40) = 6.98, p = 0.012, η_p_^2^ = 0.15) suggested that loud sounds were processed somewhat faster (517 ms) than soft sounds (525 ms). A main effect of position (F(2,80) = 4.38, p = 0.016, η_p_^2^ = 0.10) showed that sound identification was slower when the visual stimulus was positioned in the upper part of the screen (524 ms), compared to the centre (519 ms, p = 0.013, Cohen's d = 0.06). At the same time, there was no difference between the central and lower (521 ms) positions or between the upper and lower positions (all ps>0.2).

While the main effect of pitch (F(1,40) = 2.88, p = 0.098, η_p_^2^ = 0.07) did not reach significance, two two-way interactions were obtained: task by pitch (F(1,40) = 5.43, p = 0.025, η_p_^2^ = 0.12) and position by pitch (F(2,80) = 9.96, p < 0.001, η_p_^2^ = 0.20). The task by pitch interaction was driven by a significant difference between high and low pitch in the pitch task (484 vs. 496 ms, p = 0.017, Cohen's d = 0.15), with no such difference between the two pitches in the loudness task (553 vs. 552 ms, p = 1.0). The position by pitch interaction showed the expected classical cross-modal correspondence/mapping between circle position and pitch height but only for the upper position of the visual stimulus (516 vs. 532 ms, p < 0.001, Cohen's d = 0.18). The central position worked as the expected control with no modulation effect (516 vs. 521 ms, p = 1.0); more interestingly, the lower circle position showed no significant mapping either (523 vs. 519 ms, p = 1.0). Other two-way interactions were not significant (all ps>0.1).

**Figure 3.**
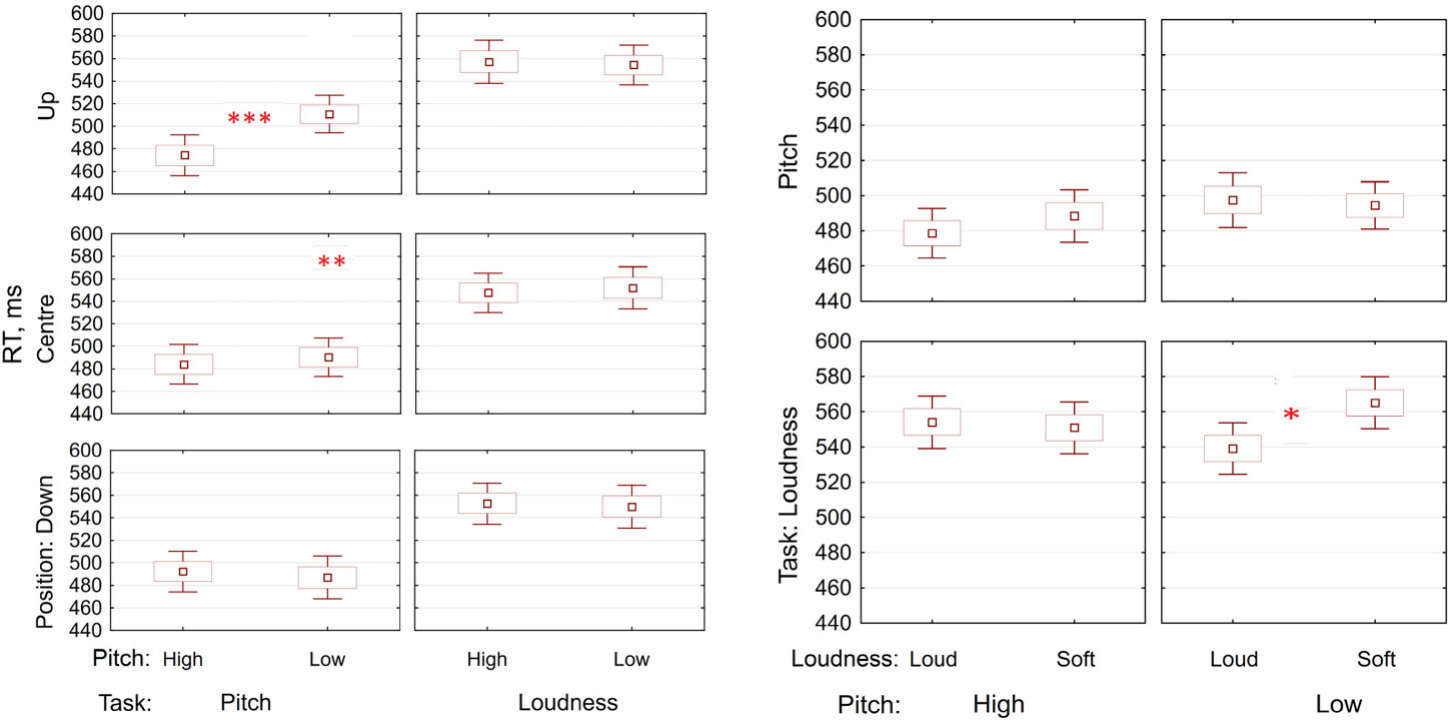
Reaction times in pitch and loudness tasks for different visual stimuli. Left panel: task X pitch X visuo-spatial position interaction. The RT of identification of high pitch was significantly faster than that of low pitch when the visually presented circle was in the upper position. A difference between RTs in upper and central positions during low pitch identification was also found. Right panel: task X pitch X loudness interaction. Identification of a soft sound was significantly impeded by low pitch. No other theoretically important difference was found. *Note*. Dots denote Means, Boxes—Mean ± SE, and vertical bars—Mean ± 95% CI *p *<* .05; **p *<* .01; ***p *<* *.001*.

Critically, rmANOVA also revealed a task X pitch X position interaction (F(2,80) = 13.53, p < 0.001, η_p_^2^ = 0.25; see Figure 3, left panel). The interaction showed that the cross-modal mapping (pitch-position) was highly dependent on the task. Pitch-position mapping was present only in the pitch identification task and only for the upper location (474 vs. 510 ms, p < 0.001, Cohen's d = 0.46) but not the lower one (492 vs. 487 ms, p = 1.0). Moreover, a significant difference between upper and central positions during low pitch identification was also found (510 vs. 490 ms, p = 0.001, Cohen's d = 0.26) which speaks of inhibitory process. The pitch-position mapping was completely absent in the loudness task (all ps = 1.0). Finally, the analysis obtained a task X pitch X loudness interaction (F(1,40) = 9.73, p = 0.003, η_p_^2^ = 0.20), illustrated in Figure 3 (right panel). Interestingly, it showed that loudness had no influence on pitch identification, however, pitch had an influence on the loudness identification: low pitch impeded identification of the soft sound (565 vs. 539 ms, p = 0.011, Cohen's d = 0.32). Other three-way interactions were not significant (all ps>0.4); neither was the four-way interaction (p > 0.07). Thus, critically, loudness showed no cross-modal correspondence effect, confirming previous result on its instability (Puigcerver et al., 2019). Most importantly, the pitch-space effect disappeared when it became task-irrelevant, suggesting non-automaticity of the cross-modal correspondence. Finally, the results demonstrated that the pitch-space correspondence effect was present only for the upper visual position and was inhibitory.

## Discussion

The present study tested the level of automaticity (as opposed to top-down attentional control) of the cross-modal audiovisual correspondence effect. To this end, we used the established model of response mapping between sound pitch and vertical visuo-spatial location. Furthermore, we tested the poorly studied subject of cross-modal audiovisual mapping between sound loudness and visual stimulus location. As an experimental approach, we modified the paradigm previously used by Evans (2020) and asked our participants to make speeded identification within auditory modality using the stimuli that varied along two dimensions (pitch and loudness). While the task-irrelevant visual stimuli (circles located at different vertical positions on the screen) and the task-relevant sound stimuli (sinewave tones with two gradations of pitch and two levels of loudness) were the same in both experimental conditions, the identification task instruction was aimed at focusing the attention on different sound features: identifying either high vs. low pitch or soft vs. loud sounds. We hypothesised that if the cross-modal correspondence is automatic we should observe cross-modal correspondence effects with no differences between the two tasks. If, however, the audiovisual correspondence is top-down controlled via the selective attention mechanisms, we should expect task-dependent modulation of the effect size. In line with the latter alternative prediction, the results showed a clear task dependence of the pitch-space effect. This effect appeared when participants were asked to do pitch identification but no similar effect was found when they were asked to identify the loudness of the same sounds (with their pitch still varied). Thus, the results revealed that selective auditory attention modulated the cross-modal effect between pitch and visual space. The manifested modulation is in accordance with a suggestion (Talsma et al., 2010) that the directed attention can influence the integration process when the stimuli are complex and when there is a competition between the stimuli which is, in our case, within auditory modality. One possible explanation for this is that pitch and loudness compete for salience within the auditory processing modality making the selective top-down regulation necessary for the cross-modal mapping to occur. However, when the attention was directed to the loudness, the pitch-space integration disappeared. Furthermore—and importantly—the study revealed no loudness-space correspondence regardless of the task (not even in the loudness identification condition), which confirms earlier observations of low reliability of audiovisual effects involving loudness and vertical space (Puigcerver et al., 2019). The latter may, in turn, stem from the absence of ecological/environmental bases for such a correspondence to arise, unlike the ecologically grounded pitch-location associations (Parise et al., 2014).

Importantly, the pitch-space effect manifested only for the upper position of the visual stimulus. The contribution of the upper space into the cross-modal effect could be explained by the perceptual and attentional asymmetry (Jóhannesson et al., 2018) in visual stimulus detection and object recognition: the upper visual field is known to have an advantage over the lower one (Thomas, & Elias, 2011; Greene et al., 2019). Hence, probably, the conflict between exogenously (visual stimulus and low pitch) driven attention was strong enough to inhibit the pitch identification process. Yet another possible interpretation could be based on data obtained by Parise et al. (2014)—it appears that there is a strong bias towards upper space in frequency-dependent sound localisation. In other words, high frequency sounds are perceived as coming from upper space which is seemingly based on the statistics of natural environment. That is, arguably, since the natural correlation between high pitch and upper space is much stronger than the natural correlation between low pitch and lower space, the ‘pitch-space conflict’ in the upper space is strong and detectable, while the ‘conflict’ in the lower space is weak and unnoticeable. Overall, the study showed behaviourally for the first time that selective attention makes a cross-modal effect disappear, which speaks in favour of a possible attentional component in its generation and thus, theoretically, in multisensory integration (Talsma, 2015; Tang, et al., 2016). Obviously, the interaction between attention and cross-modal correspondence is situation-dependent. We tested only two attended auditory dimensions and only one (vertical) visual dimension, so a consideration for future research would be to vary dimensions in visual and other modalities, as well as other stimulus properties, in order to determine the extent of generalisation and stability of the interactions between attention and cross-modal correspondence.

## Supplemental Material

sj-xlsx-1-ipe-10.1177_20416695221095884 - Supplemental material for Look and ye shall hear: Selective auditory attention modulates the audiovisual correspondence effect Click here for additional data file. Supplemental material, sj-xlsx-1-ipe-10.1177_20416695221095884 for Look and ye shall hear: Selective auditory attention modulates the audiovisual correspondence effect by Armina Janyan, Yury Shtyrov, Ekaterina Andriushchenko, Ekaterina Blinova and Olga Shcherbakova in i-Perception
